# Physiological and behavioral responses of house sparrows to repeated stressors

**DOI:** 10.7717/peerj.4961

**Published:** 2018-06-06

**Authors:** Brenna M.G. Gormally, Jessica Wright-Lichter, J. Michael Reed, L. Michael Romero

**Affiliations:** Department of Biology, Tufts University, Medford, MA, United States of America

**Keywords:** Chronic stress, Corticosterone, Immune capacity, Reactive scope, Allostasis, Neophobia

## Abstract

Despite decades of research, we still lack a complete understanding of what factors influence the transition of the necessary and adaptive acute stress response to what has become known as chronic stress. This gap in knowledge has illuminated the necessity for studies that examine the thresholds between these two sides of the stress response. Here, we determine how repeated exposure to acute stressors influences physiological and behavioral responses. In this repeated measures study, house sparrows (*Passer domesticus*) were exposed to a chronic stress protocol. We took physiological and behavioral measurements before, during, and after the protocol. Blood samples were used to assess four aspects of hypothalamic-pituitary-adrenal (HPA) axis function: baseline corticosterone, stress-induced corticosterone, negative feedback, and the maximal capacity to secrete corticosterone. We also assessed bacterial killing capacity and changes in uric acid concentration. Neophobia trials were used to assess behavioral changes throughout the protocol. We found no significant changes in HPA axis regulation in any of the four aspects we tested. However, we found that uric acid concentrations and neophobia significantly decreased after only four days of the chronic stress protocol, while bacterial killing capacity did not decrease until after eight days of exposure. These results indicate that different components of the stress response can be impacted by chronic stress on different timescales. Our results further indicate the importance of assessing multiple aspects of both physiology and behavior in order to understand how exposure to chronic stress may influence ability to cope with future challenges.

## Introduction

In recent decades, the stress response has become widely studied across many disciplines including ecology (e.g., Bonier et al., 2009; Brearley et al., 2012; Crespi et al., 2013), conservation biology (e.g., Fefferman & Romero, 2013; Partecke, Schwabl & Gwinner, 2006; Walker, Dee Boersma & Wingfield, 2006), and field endocrinology (e.g., Bauer et al., 2014; Love et al., 2016; Schoech, Bowman & Reynolds, 2004). Many of these studies focus on how physiological and behavioral systems work in tandem to enable an animal to cope with stressful stimuli (Bennett et al., 2016). Other studies consider the negative implications of chronic stress, which can occur when the very systems that help an organism survive become overworked (Cyr et al., 2007; McCormick, Shea & Langkilde, 2015; Pavlides, Nivón & McEwen, 2002). For example, rapid, acute increases in sympathetic nervous system activity via catecholamines is crucial for the upregulation of essential survival systems during a stressor (Nephew & Romero, 2003); however, chronic elevations can lead to hypertension, stroke, insulin resistance, and hypertriglyceridemia (Rupp, 1999). Despite the wide array of studies, we still lack a complete understanding of how these responses interact and what factors influence the transition of acute, beneficial stress responses to those characterized as chronic or harmful (Romero et al., 2015). To begin teasing apart these factors, researchers need to better understand whether identifiable thresholds exist between these types of responses. The purpose of this study was to push house sparrows (*Passer domesticus*) to this threshold in order to identify key transition points in physiological and behavioral indices.

The stress response involves several behavioral and physiological systems (e.g., Romero & Wingfield, 2016; Sapolsky, Romero & Munck, 2000). We focused on four of those responses in this study—hypothalamic-pituitary-adrenal (HPA) axis regulation, immune killing capacity, metabolism, and neophobic behavior. The HPA axis is the primary focus of many stress studies, with particular attention paid to the glucocorticoids cortisol and corticosterone (CORT) (Romero & Wingfield, 2016). With receptors in nearly every tissue (Lattin et al., 2015), CORT has extremely wide-reaching effects (Ballard et al., 1974; Lattin et al., 2012b). Acute activation of the HPA axis and elevation of CORT induces the transcription of proteins that broadly upregulate essential, and suppress nonessential, survival systems (Sapolsky, Romero & Munck, 2000). What has become increasingly obvious, however, is that CORT concentrations do not have a clear relationship with chronic stress; symptoms of chronic stress do not necessarily develop in tandem with elevated CORT concentrations (Dickens & Romero, 2013). Other aspects of HPA axis regulation are also important, including total physiological capacity of CORT production as well as the shut-down of CORT release via negative feedback (Dallman & Bhatnagar, 2010; Dickens, Delehanty & Romero, 2010).

CORT helps regulate downstream effects of the stress response, including metabolic, immunological, and behavioral effects (Romero & Wingfield, 2016). CORT is primarily recognized for the metabolic processes it either directly or permissively promotes. These include gluconeogenesis (Romero & Wingfield, 2016) and glycogenolysis (Nonogaki, 2000). These catabolic processes increase the breakdown of proteins, and thus elevations in both blood glucose and uric acid—the major end product of nitrogen catabolism—are seen following acute CORT increases (Lin, Decuypere & Buyse, 2004a). Uric acid is also an important antioxidant (Ames et al., 1981) and it’s therefore been suggested that stress-induced changes may be related to maintaining redox balance (Cohen, Klasing & Ricklefs, 2007). In addition to these metabolic roles, CORT is hypothesized to both augment and inhibit immunity and inflammation on different timescales; acute activation can enhance immunity while chronic activation can lead to immunosuppressive effects (Dhabhar, 2002; Dhabhar & McEwen, 1999; Dhabhar & McEwen, 1997). Finally, CORT can influence the expression of several behaviors, including elevated activity (Breuner, Greenberg & Wingfield, 1998), survival behavior (Astheimer, Buttemer & Wingfield, 1992), and neophobia—the fear/avoidance of novel objects (Lendvai, Bókony & Chastel, 2011).

While acute activation of the stress response is important, chronic activation, either through exogenous CORT administration or psychological or physical stress, has been shown to dysregulate many of these functions. HPA axis function itself can become altered during periods of chronic stress. Baseline CORT can become elevated (Dickens & Bentley, 2014; Lattin, Pechenenko & Carson, 2017; Lattin et al., 2012a; Love, Lovern & DuRant, 2017) as well as reduced (Cyr et al., 2007; Rich & Romero, 2005), while stress-induced levels tend to decrease (Cyr et al., 2007; Lattin et al., 2012a) during laboratory-induced chronic stress. Regulation of the HPA axis can also be affected, as reflected in weakened (Dickens, Earle & Romero, 2009b) or strengthened (Lattin et al., 2012a) negative feedback, but sustained or strengthened maximum capacity (e.g., Dickens, Delehanty & Romero, 2009a; Lattin et al., 2012a). Many of these effects are dependent on crucial factors including species and life history stage. Chronic stress can also cause significant weight loss (Bartolomuccia et al., 2004; Cyr et al., 2007). Uric acid has been shown to increase under chronic corticosterone administration in broiler chickens (*Gallus gallus domesticus*) (Lin, Decuypere & Buyse, 2004b), but remain unchanged under chronic psychological stress conditions in European starlings (*Sturnus vulgaris*) (Awerman & Romero, 2010). Additionally, the immune system has been shown to be suppressed by chronic stress in some studies (Dhabhar & McEwen, 1997; DuRant et al., 2016a; Martin et al., 2012), while other studies have found maintenance of immune function (DuRant et al., 2016b; Love, Lovern & DuRant, 2017). Finally, chronic stress has been shown to decrease activity in open field tests (Katz, Roth & Carroll, 1981) and increase neophobia (Wisłowska-Stanek et al., 2016) in rodents. As is clear from the above list of previous studies, physiological and behavioral parameters rarely change in consistent ways across different studies and species. However, few studies have measured multiple parameters concurrently. In the present study, we chose to measure indices of HPA axis regulation, metabolism, immune function, and behavior in an attempt to reach a more comprehensive and conclusive phenotype of chronic stress in a single species.

**Figure 1:**
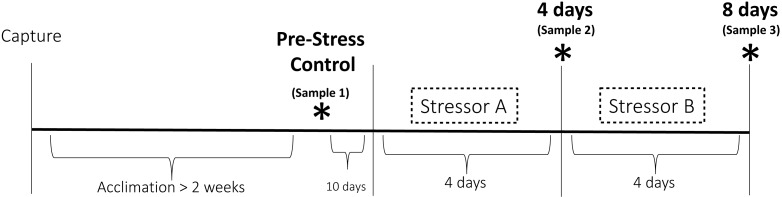
Experimental design of the chronic stress protocol. Birds were randomly assigned to one of two treatment groups. One group received stressor A (cage rolling) for four days followed by stressor B for four days (cage tapping). The other group was exposed to these stressors in the opposite order. Three sets of physiological and behavioral samples (denoted by the asterisks) were taken before (Pre-Stress Control), during (four days), and after (eight days) the stress period.

The goal of this study was to expose house sparrows to repeated acute stressors over the course of several days to push them just to the edge of experiencing serious symptoms of chronic stress. Prior studies have shown that different aspects of HPA axis function and regulation are significantly altered after eight to ten days of simulated chronic stress in laboratory and field settings (Cyr et al., 2007; Cyr & Romero, 2007; Dickens, Earle & Romero, 2009b; Rich & Romero, 2005). While we know that CORT becomes affected on this timescale, we do not yet know how other components of the stress response are affected. Furthermore, it has become clear that CORT is not the best indicator of when an animal might be a risk of experiencing negative symptoms of chronic stress (Dickens & Romero, 2013). Therefore, in this study we sought to test how repeated exposure to stressors influences not only HPA axis regulation but also immune capacity, metabolism, and behavior in house sparrows. This was accomplished by exposing house sparrows to eight days of stressors—four days of one stimulus followed by four days of another stimulus (Fig. 1). We hypothesized that the effects of exposure over the eight-day period would become amplified over time. In other words, the initial four-day exposure would reduce the birds’ ability to cope with future challenges (the final four-day exposure). Therefore, we expected to see more serious changes in the measured indices at the final sample point relative to that taken in the middle of the protocol. We predicted that, based on previous studies referenced above, this would not be reflected in altered HPA axis regulation (reduced baseline and stress-induced CORT, disrupted negative feedback, decreased capacity), but would be reflected in immunosuppression, elevated uric acid, and enhanced neophobic responses.

## Materials and Methods

### Experimental design

To test the physiological and behavioral effects of repeated exposure to stressors, 22 wild free-living house sparrows (12 females, 10 males) were caught June 1–3 2016 in Medford, MA, USA. Individual birds were randomly assigned to one of two treatment groups and housed in cages in separate rooms according to this distinction. Randomization occurred such that each group contained both males and females and such that no group contained birds that were all captured on the same day. Birds were housed in pairs and/or in view of other birds in cages (45 cm × 37 cm × 33 cm) and kept on a natural 15L:9D light cycle. An acclimation period occurred for at least 2 weeks during which birds were disturbed only for routine husbandry. This period was determined based on previous research involving house sparrows, which showed hormonal acclimation occurring after 2 weeks in captivity (Fischer, Wright-Lichter & Romero, 2018).

This study was a repeated measures design in which the responses of individual birds during the experiment were compared to pre-stress samples. At the end of the initial 2-week acclimation period, a pre-experiment blood sample was taken (“Pre-Stress Control” in Fig. 1). This sample served as the control to which the responses to chronic stress were compared. We did not include a separate control group of birds that did not experience the chronic stress treatment because prior research has shown that no significant hormonal changes occur to house sparrows following acclimation (Dickens et al., 2009c; Lattin & Romero, 2014; Rich & Romero, 2001). The chronic stress protocol commenced 10 days after the end of the acclimation period. Birds in treatment group A received stressor A for four days followed by four days of stressor B, while birds in treatment group B received the stressors in the opposite order (Fig. 1). This was to ensure that the results were not stimulus or order-dependent. Stressor A (cage rolling) involved the cages being placed on lab carts and being gently rolled back and forth for 30 min twice per day. During stressor B (cage tapping), one person entered the experimental room every 2.5 min and tapped on the cages; this persisted for 30 min twice per day. The birds experienced these stressors at unpredictable times throughout the day, but always twice per day. Repeated variable application of acute stressors has been shown used to elicit physiological and behavioral changes consistent with chronic stress in many species (e.g., Cyr et al., 2007; Rich & Romero, 2005), including using both of these stimuli in previous studies involving house sparrows (DuRant et al., 2016a). Each stressor was presented for 4 days; a time period chosen based on previous studies that suggested physiological changes indicative of chronic stress occur over eight to ten days of stimulus presentation (Cyr et al., 2007; Rich & Romero, 2005).

Animals were collected under a Massachusetts state collection permit and all experiments were conducted with the approval of the Tufts Institutional Animal Care and Use Committee and in compliance with the Guidelines for Use of Wild Birds in Research (Fair, Paul & Jones, 2010).

### Plasma sampling

Blood samples were taken at three time points to assess HPA axis functioning, immune capacity, and changes in uric acid concentrations (Fig. 1). The control sample was taken 10 days prior to exposure to the experimental stressors and at least two weeks after initiation of captivity. We decided on this separation between the control sample and the start of the stress protocol to remain under the ethical requirement for volume of blood for sampling. A second sample (“4 days” in Fig. 1) was taken after four complete days of the first stressor and a third and final sample (“8 days” in Fig. 1) was taken after the entire eight-day protocol. On each of these days, a series of blood samples was taken starting at 9:00am. Baseline samples were taken within 3 min of entering the room (Romero & Reed, 2005). Birds were then placed in an opaque cloth bag for 30 min, after which a stress-induced sample was taken. At this time, 1 mg/kg dexamethasone (12.5 µL; DEX; Phoenix Pharmaceuticals, Inc., St. Joseph, MO, USA), a synthetic glucocorticoid, was injected into the pectoralis muscle (30 G, BF PrecisionGlide™ Needle, Becton, Dickinson and Company, Franklin Lakes, NJ, USA) to assess efficacy of negative feedback in the HPA axis. Birds were sampled again 90 min after DEX injection, then 100 IU/kg ACTH (10 µL; Sigma Aldrich, St. Louis, MO, USA; Catalog No. A6303) was injected as before to assess the maximum capacity of the HPA axis. The final blood sample was taken after an additional 15 min of restraint. All blood samples were taken from the alar vein and collected in heparinized capillary tubes. Samples were stored on ice until processing, which occurred no more than 5 h after sampling. Centrifugation at ∼1,200 g for 10 min (Centrific Model 225, Fisher Scientific, Pittsburgh, PA, USA) was used to separate plasma, which was frozen at −20 °C until assay. Separate aliquots were created from the baseline samples in order to conduct CORT, bacterial killing, and uric acid assays.

### Corticosterone assays

Radioimmunoassays (RIA) were used to quantify CORT concentrations in the baseline, stress-induced, negative feedback, and Max plasma samples (Wingfield, Vleck & Moore, 1992). Briefly, steroids were extracted from plasma samples (13.3 ± 5.9 µL) using dichloromethane, dried under N_2_ gas, and then rehydrated with 550 µL phosphate-buffer saline with gelatin. A standard curve was created and the RIA was run using the antibody B3-163 (Esoterix, Calabasas Hills, CA, USA). Assay sensitivity was 1 ng/ml; baseline and negative feedback samples were often below the level of detection (20 samples) and therefore assigned this floor value. The inter and intra-assay CVs were 7.6% and 3.5% respectively.

### Bacterial killing assays

Baseline plasma samples were also used for *in vitro* analyses of bacterial killing capacity. All samples were assayed within 10 days of sampling as longer periods of freezing have been shown to dramatically affect killing (Liebl & Martin, 2009). This assay assesses the proficiency of plasma antibodies and proteins to combat a pathogen and it has been validated for house sparrows (Liebl & Martin, 2009). Briefly, 1.5 µL of plasma was diluted with 34.5 µL PBS and was incubated with 12.5 µL of *Escherichia coli* (10^4^ bacteria/mL; ATCC^®^ 8739) at 37 °C for 30 min. Then, 250 µL of tryptic soy broth (TSB) was added to each tube, followed by an additional 12-hour incubation at 37 °C. Positive controls with only *E. coli* and TSB were also created. Finally, a NanoDrop spectrophotometer (NanoDrop 2000, Thermo Fisher Scientific, Waltham, MA, USA) was used to quantify absorbance of each sample at 300 nm. Killing capacity was calculated as 1 –(absorbance of sample / absorbance of positive control). All samples were run in triplicate when possible; only 4 samples out of all those that were assayed had to be run in duplicate. The inter-assay CV, based on the positive controls, was 11.5%.

### Uric acid fluorometric assay

Baseline plasma samples were used to quantify uric acid using the Amplex^®^ Red Uric Acid/Uricase Assay Kit (Molecular Probes, Eugene, OR, USA). This assay relies on the conversion of uric acid to hydrogen peroxide, which subsequently reacts with Amplex^®^ Red reagent in the presence of horseradish peroxidase (HRP) to form a red-fluorescent product. Briefly, plasma samples were diluted with 200 µL of the provided reaction buffer and a uric acid standard curve was generated using the provided reagents. To have enough reagents to run all samples, the Mastermix was slightly altered to include 150 µL of Amplex^®^ Red reagent and 85 µL of the reaction buffer. To account for this change, spectrophotometric readings were taken at 30 and 45 min at 560 nm. No statistical differences were found between these samples and therefore all further analysis was completed using the 30-minute sample. A negative control, lacking uric acid, and a maximum-reading positive control were also included on the 96-well plates. Four separate assays were completed and samples were run in triplicate. The inter-assay CV from the positive controls was 7.3%.

### Neophobia and perch hopping trials

In addition to the physiological data gathered from plasma sampling, changes in behavior were assessed by measuring the birds’ responses to novel objects. Three neophobia trials were conducted prior to any stimuli, after five days of the chronic stress protocol, and after seven days of the chronic stress protocol. Because blood and behavioral samples could not be taken the same day, we were limited which days the neophobia trials could occur. Beginning five days prior to the first neophobia trial, food dishes were removed each night (within 1 h before lights off) and replaced each morning (within 1 h after lights on) to acclimate the birds. At this time, the cage liners were also replaced to remove any excess food was removed from the bottom of each cage; this was to ensure that birds would reliably return to their food in the morning.

On the night directly before a trial, food dishes were removed and opaque blinders were placed between each cage to prevent the birds from seeing the neighboring trial, which could alter their own behavior (Fryday & Greig-Smith, 1994). The following morning, food was replaced with one of three possible novel objects—a colored plastic egg in the dish, a red ribbon across the dish, or a food dish painted red. All objects had been used previously in studies involving avian species and shown to elicit a neophobic response (Fischer & Romero, 2016). Behavior was recorded for 20 min after initial food presentation and then the novel objects and blinders were removed. Each individual’s latency to feed was recorded. Feed latency was defined as the time between replacing the food and the bird visibly feeding. Additionally, perch hopping was calculated during 15 min of the same trial. The first 5 min were discounted to avoid activity that occurred due to the presence of the experimenter replacing the food dish. A perch hop was defined as movement throughout the cage, typically between the perch, water dish, cage, and/or ground.

Three control trials were also conducted (one each day) prior to each neophobia trial to ensure that the stress treatments did not affect the birds’ normal feeding behavior. During these control trials, food dishes were returned in the morning without a novel object. Neophobia and control trials were conducted on days separate from blood sampling.

### Data analysis

All statistical analyses were conducted in RStudio (RStudio Team, 2015). Before performing any linear mixed effects models or ANOVAs, the data were checked for heteroscedasticity using Bartlett’s test. In the event of a violation, data were log-transformed; this was the case with baseline and stress-induced CORT as well as with perch hopping.

#### Group and sex effects

Before conducting further analyses, we tested whether there was an effect of group or sex in any of the datasets. This was done in order to check if differences in sex or stimulus order (whether cage rolling or cage tapping was experienced first) caused significant effects to physiological or behavioral responses. We used linear mixed effects models (LMM) with individual bird identity as a random effect and group and sample as main effects in separate models (lme4 package; Bates et al., 2015). The ‘Anova’ function in the car package (Fox & Weisberg, 2011) generated results for Type III Wald *F*-tests with Kenward-Roger adjusted degrees of freedom. Interactions were checked using the same method, but with an interaction term between group or sex and sample.

#### Hormone data

Four baseline samples (*n* = 62) and three negative feedback samples (*n* = 63) did not contain enough plasma to be run. One sample from the stress-induced data was dropped because that bird did not mount an acute stress response to the restraint stimulus and a second sample did not contain enough plasma (*n* = 64). The different CORT responses measured here (baseline, stress-induced, negative feedback, and maximal capacity) play complicated activation, inhibition, permissive, and preparative roles and interact with different physiological systems (Romero, 2004; Sapolsky, Romero & Munck, 2000). Because these are distinct physiological responses often regulated by different receptors, four separate models were used to analyze changes in each type of CORT sample across each of the three sampling points. However, bonferonni adjustments were used, if necessary, to account for non-independence of the dependent variables. The efficacy of negative feedback was calculated as the percent decrease in CORT from stress-induced levels to those after injection of DEX. LMMs with individual bird identity as a random effect, were used to analyze baseline, stress-induced, negative feedback, and maximum CORT responses (lme4 package; Bates et al., 2015). All models were visually checked for model assumption violations using residual plots.

Finally, for each bleed type, the ‘Anova’ function was used to run ANOVAs with sample as a main effect and bird ID as a random effect. In the event of significant result, Tukey’s multiple comparison post-hoc tests were run using the ‘glht’ function in the multcomp package (Hothorn, Bretz & Westfall, 2008).

#### Immune capacity and uric acid concentrations

One sample did not contain enough plasma to be run in the BKA (*n* = 65) while two samples did not contain enough plasma to be run in the uric acid assay (*n* = 64). A single LMM was used to assess changes in killing capacity and uric acid concentrations over the course of the chronic stress protocol; again, individual bird identity was included as a random effect. Tukey’s multiple comparison post-hoc analyses were conducted as indicated above.

#### Behavioral data

Birds that failed to approach the novel object in the allotted time were assigned a ceiling value of 20 min. Interactions between feed latency and novel object were assessed with separate LMMs and a final LMM was run to compare feed latencies across the sampling protocol. Perch hops were counted during the last 15 min of the neophobia videos. The first 5 min were discounted to avoid confounding effects of experimenter presence. All videos were coded by the same person. The movements of two birds were obscured from where the camera was placed and one sample from an additional bird was removed from analysis because they did not display any perch hopping in the allotted time, making log transformation impossible (*n* = 59). Interactions between total perch hops and group, sex, and object were checked with individual LMMs and again a separate LMM was completed to compare activities across sampling periods. Separate models were used to assess changes in the control trials for both the feed latency and perch hopping data sets.

## Results

### Group and sex effects

There was no main effect of group in the datasets for stress-induced, negative feedback, and maximum capacity CORT, bacterial killing, uric acid, or neophobia datasets (*p* > 0.10 for all tests). There were also no significant interactions between group and sample (*p* > 0.54 for all tests). Therefore, stimulus order was removed from any further analyses of those datasets. We conducted the same check for sex effects and interactions and found none (*p* > 0.06 for all main effects tests and *p* > 0.28 for all interaction tests); sex was therefore removed from any further analyses in all datasets, except for the perch hopping data where we did detect a significant effect of sex.

### Corticosterone responses

**Figure 2:**
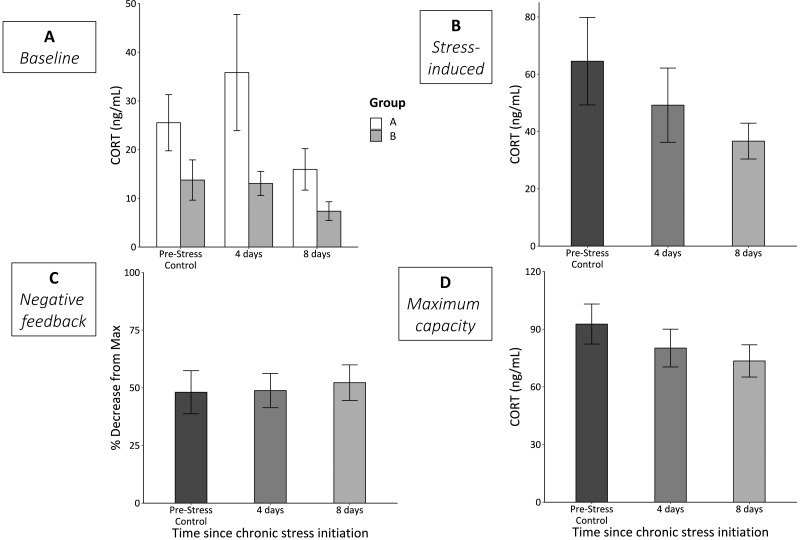
CORT responses before (Pre-Stress Control), during (4 days), and after (8 days) the chronic stress protocol. (A) Baseline samples were taken within 3 min of entering the experimental room. White bars represent group A (birds experienced cage rolling first) and grey bars represent group B (birds experienced cage tapping first). While there was a significant main effect of sex (*p* = 0.03), neither group A (*p* = 0.33) nor group B (*p* = 0.23) experienced significantly changes baseline CORT. (B) Stress-induced CORT samples were taken 30 min after restraint in an opaque cloth bag and did not significantly change (LMM *p* = 0.25). (C) Efficacy of negative feedback remained constant throughout the protocol (LMM *p* = 0.93). (D) Physiologically maximum levels of CORT taken after injection of ACTH did not change (LMM *p* = 0.3). Error bars represent ±SEM. Note the different *y*-axes.

Group—the order that the stimuli were experienced in—significantly affected baseline CORT (*F*_1,60_ = 5,73, *p* = 0.03; Fig. 2A). Birds in group A (experienced cage rolling first) had higher baseline CORT at each sample point. However, even when analyzed separately, we did not detect statistically significant differences in either group A (*F*_2,27_ = 1.18, *p* = 0.33) or group B (*F*_2,29_ = 1.57, *p* = 0.23) across the chronic stress protocol. We also did not detect any statistically significant differences in CORT levels at any of the other three bleed types (stress-induced, *F*_2,61_ = 1.42, *p* = 0.25; negative feedback, *F*_2,60_ = 0.07, *p* = 0.93; maximum capacity, *F*_2,63_ = 1.24, *p* = 0.30) over the course of the chronic stress protocol (Figs. 2B–2D).

### Immune capacity

**Figure 3:**
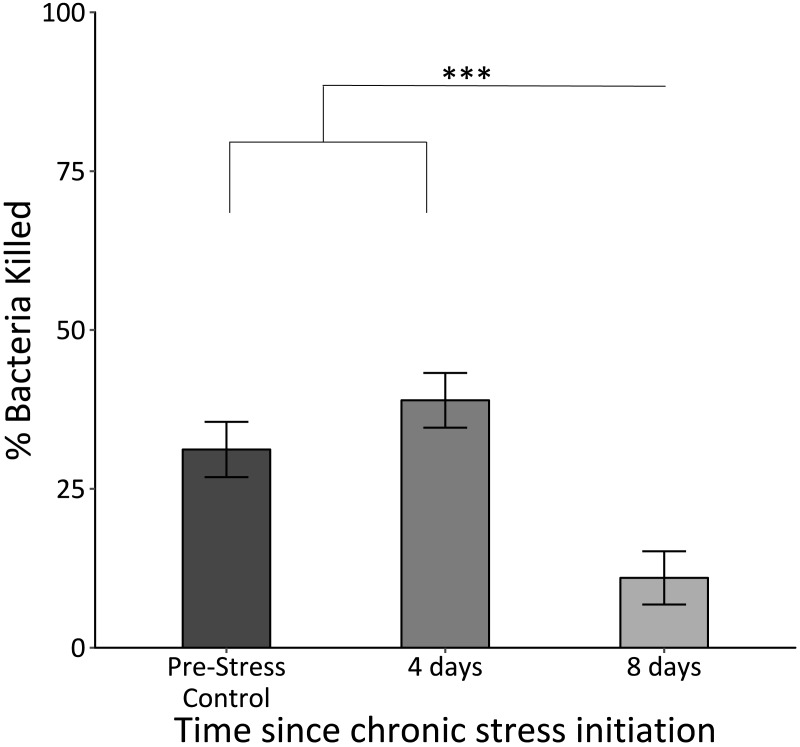
*In vitro* bacterial killing capacity of plasma samples from birds before (Pre-Stress Control), during (four days), and after (eight days) the chronic stress protocol. Killing capacity remained unchanged after four days of the chronic stress protocol, but became significantly reduced after eight days. Triple asterisk denotes sample 3 different from samples 1 and 2 with a significance of *p* < 0.0001. Error bars represent ± SEM.

The killing capacity of samples taken at the final time point was significantly lower than those for those at the control and second time point (*F*_2,62_ = 19.7, *p* < 0.0005; Fig. 3).

### Uric acid

**Figure 4:**
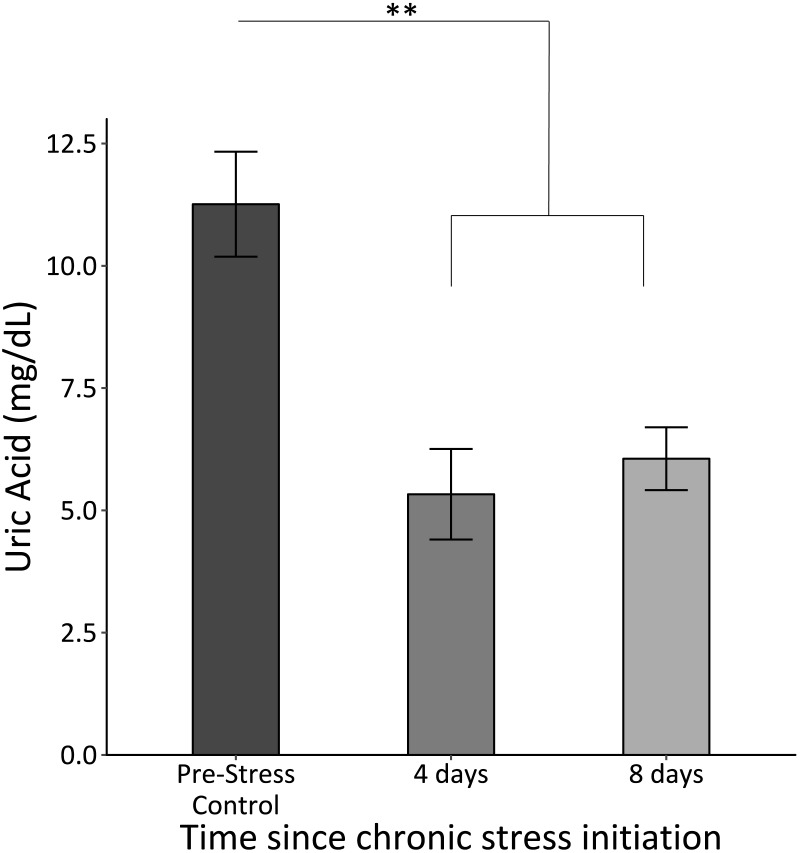
Uric acid concentrations before (Pre-Stress Control), during (four days), and after (eight days). Baseline uric acid significantly decreased after four days of chronic stress. Double asterisk denotes significance at *p* < 0.001. Error bars represent ± SEM.

Uric acid significantly decreased once the chronic stress protocol was initiated and remained low (*F*_2,61_ = 12.8, *p* < 0.0005; Fig. 4).

### Neophobia

**Figure 5:**
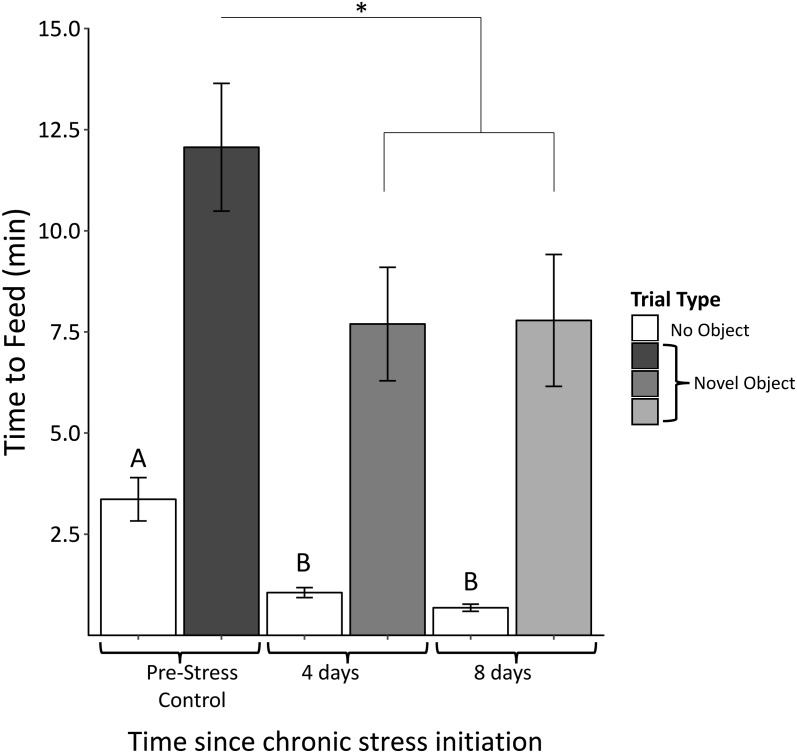
Time to feed when novel objects are present before (Pre-Stress Control), during (four days), and after (eight days) the chronic protocol. Feed latency decreased significantly after 4 days of chronic stress. White bars indicate control samples (no novel object present) taken within two days of neophobia trials. Control and neophobia trials were analyzed independently. Asterisk denotes *p* < 0.05. Different letters indicate significance at *p* < 0.0001. Error bars represent ±SEM.

We found no significant interactions between object type (red dish, red ribbon, plastic egg) and sample (*F*_4,61_ = 0.58, *p* = 0.68). There was, however, a significant main effect of object (*F*_2,63_ = 3.94, *p* = 0.03). Regardless of object type, feed latency decreased significantly after 4 days of the chronic stress protocol (*F*_2,63_ = 4.03, *p* = 0.03; Fig. 5). Feed latencies also significantly decreased during control trials as the chronic stress protocol progressed (*F*_2,63_ = 25.9, *p* < 0.00001; Fig. 5, white bars).

### Perch hopping

**Figure 6:**
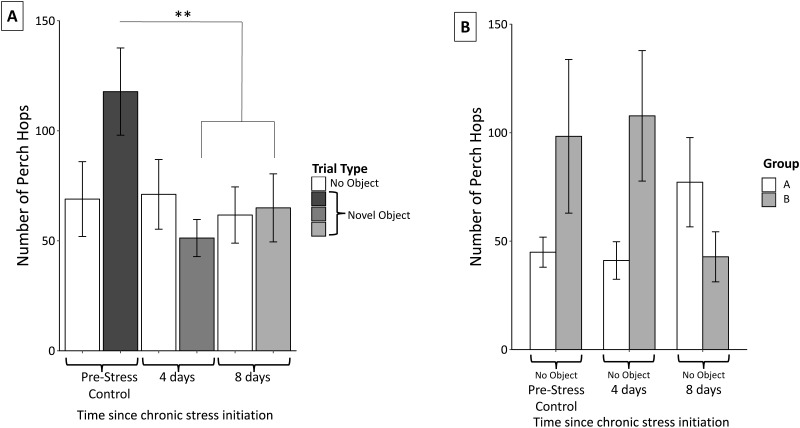
Changes in perch hopping during the chronic stress protocol. (A) Number of perch hops when novel objects are present before (Pre-Stress Control), during (four days), and after (eight days) chronic stress protocol. Perch hopping significantly decreased after four days of the chronic stress protocol. White bars indicate control samples (no novel object present) taken within two days of novel object trials. Control and neophobia trials were analyzed independently. (B) A significant interaction was found between group (whether the birds received stressor A or B first) and sample. This was driven by birds in group A increasing and those in group B decreasing perch hopping at the final sample point. Double asterisk denotes *p* < 0.001. Error bars represent ±SEM.

Perch hopping was not significantly affected by neophobic object (*F*_2,56_ = 1.02 *p* = 0.37) nor were there significant interactions between object and perch hops (object, *F*_4,54_ = 0.37, *p* = 0.83). Perch hopping significantly decreased after the initiation of the stress protocol and remained reduced through the remainder of the protocol (*F*_2,56_ = 11.16, *p* < 0.0005; Fig. 6A).

Perch hopping remained constant during the control trials. We did detect a significant main effect of sex; females were more active than males (*F*_1,57_ = 6.23, *p* = 0.02), however neither females (*F*_2,30_ = 0.27, *p* = 0.77) nor males (*F*_2,23_ = 0.16, *p* = 0.86) significantly changed their activity during these control trials. We also found a significant interaction between group and perch hopping (*F*_6,52_ = 7.52, *p* < 0.005) during the control trials when no novel objects were present. Further inspection of graphs revealed that this interaction was driven by birds in group A (experienced cage rolling first) increased their perch hopping at the final sample point, while those in group B (experienced cage tapping first) decreased their perch hopping (Fig. 6B).

## Discussion

This study examined the physiological and behavioral changes that occurred in house sparrows in response to a series of repeated acute stressors, simulating a chronic stress protocol. The findings indicate that symptoms of chronic stress can appear at three different timescales when measured in four different indices. While eight days was not long enough to elicit significant changes in HPA axis function and regulation, birds experienced substantial changes to uric acid levels and behavior after only four days of the chronic stress protocols and to immune capacity after eight days. These data are significant because they further indicate that CORT may not always the best indicator of when an animal is entering homeostatic overload.

### Effects of chronic stress on HPA axis regulation

We found no significant changes in any of the four measurements of HPA axis regulation and responsiveness across the three sampling time points (Fig. 2). The lack of change in baseline CORT levels was not entirely surprising as a previous study using house sparrows and a rotating, repeated psychological stress protocol found no significant changes (Lattin & Romero, 2014). Interestingly, there was a main effect of group on baseline CORT. Despite randomizing the birds into groups A and B, group A birds tended to have higher baseline CORT, even prior to experiencing any stimuli (Fig. 2A). Regardless, when analyzed separately, we did not detect a statistically significant change in CORT over the course of the experiment; however, both groups appeared to exhibit a similar downward trend by day 8 (Fig. 2A). Additionally, stress-induced CORT levels were also trending down relative to the control levels at the start of the experiment; it is likely that had the experiment continued, we would have found statistically significant decreases, as shown in prior studies (Cyr et al., 2007; Cyr & Romero, 2007; Dickens, Earle & Romero, 2009b; Rich & Romero, 2005). We want to stress that our primary goal was not to induce significant changes in HPA axis function and regulation, but rather to push animals to the edge of when these changes might begin. Thus, this protocol was effective at pushing animals to the edge of experiencing homeostatic overload, at least as measured by metrics of the HPA axis. It is also unlikely that these results suggest acclimation or habituation to the chronic stress protocol since the stressors were encountered at random and unpredictable times throughout the day (see Rich & Romero, 2005).

Surprisingly, we found no changes in negative feedback strength or maximum CORT production throughout the course of the experiment (Fig. 2). We expected that negative feedback strength and production capacity would change as wear-and-tear increased throughout the protocol. Previous studies involving avian species have reported changes in both these measurements due to different types of chronic stress. For example, captivity combined with translocation of chukar (*Alektoris chukar*) significantly reduced feedback strength (Dickens, Delehanty & Romero, 2009a). In addition, HPA axis maximum capacity increased in house sparrows due to initial transference to captivity during two life history stages (late breeding and molt) (Lattin et al., 2012a). These differences suggest that experimental conditions play a role in the physiological changes that occur due to chronic stress. Though translocation, captivity, and a rotating, repeating, psychological stress protocol are each considered long-term “chronic” stressors, animals likely interpret these stressors differently; it is therefore plausible that the physiological changes that result from these stimuli are unique.

### Immunological effects of chronic stress

Our results indicate that immune capacity was severely affected in chronically stressed birds. While birds maintained an *in vitro* immune response after the first bout of stressors, the building wear-and-tear throughout chronic stress protocol exposure induced immunosuppression following the second bout (Fig. 3). This finding was consistent with our predictions based on evidence that chronic stress leads to immunosuppression while acute stress can lead to immunoenhancement (Dhabhar, 2002; Glaser et al., 1987; Martin, 2009; McEwen, 1998). Furthermore, the data presented here are consistent with a previous study in which elevating allostatic load through topical CORT application slowed house sparrows’ healing of a superficial wound (DuRant et al., 2016a). What we find particularly intriguing about these results is that four days of the chronic stress protocol is not enough to induce changes in immune capacity, as it is in the case of uric acid and behavior. The initial maintenance of bacterial killing capacity is possibly indicative of an initial enhancement of immune function. It is possible that during the initial, “short-term” 4 days of the chronic stress protocol, immune function is actually enhanced. By the time the second sample is taken however, immune function is returning to original levels, before falling even further upon exposure to the second set of stressors. Prior studies have indicated the complex balance between stress and immunity with short-term exposure enhancing and long-term exposure suppressing (Dhabhar & McEwen, 1999; Martin, 2009).

We believe that this final decrease in *in vitro* immune function is representative of the birds transitioning into a “chronically stressed” state, or what the reactive scope model terms homeostatic overload (Romero, Dickens & Cyr, 2009). The fact that this is happening after approximately 8 days of the chronic stress protocol supports prior findings that have investigated the physiological changes during chronic stress (Cyr et al., 2007; Cyr & Romero, 2007; DuRant et al., 2016a; Rich & Romero, 2005). While we recognize that this is a single measure of immune function and relies on only one kind of pathogen, the BKA has been found to be correlated with other immune parameters and assays in avian species, including lipopolysaccharide challenge, phagocytosis assay, and acute phase proteins (Millet et al., 2007).

### Metabolic changes during chronic stress

Interestingly, uric acid concentrations decreased immediately during the chronic stress protocol following the initial stimulus (Fig. 4). This was contrary to predictions based on previous studies that indicated that chronic corticosterone exposure or psychological or physical stress lead to elevations in uric acid in avian species (Azad et al., 2010; Lin, Decuypere & Buyse, 2004a; Lin, Decuypere & Buyse, 2004b). While a prior survey of nearly 100 bird species found that uric acid concentrations predominately fall following the acute stress response (Cohen, Klasing & Ricklefs, 2007), fewer studies have examined uric acid changes due to chronic stress, independent of CORT administration. We predicted that basal uric acid concentrations would increase over time because as birds experienced the chronic stress protocol, their metabolism would increase, thus leading to more uric acid as a byproduct of purine metabolism. However, these previous studies specifically relied on elevations of CORT to induce elevations in uric acid, whereas our stress protocol resulted in no changes in baseline CORT. Furthermore, previous studies report that uric acid is an important and potent antioxidant in avian species; one study specifically indicated that reduced levels of uric acid correlate with increased oxidative stress (Klandorf et al., 2001). In the present study, reduced uric acid concentrations could be an indication that the redox balance becomes upset within four days of the chronic stress protocol. In other words, the chronic stress protocol caused the house sparrows to use up their uric acid stores, which are not replenished, or at least not replenished fast enough to compensate for ongoing uric acid catabolism. This change suggests that birds may not have the same antioxidant buffer under chronic stress conditions as they do under “normal” conditions.

We find it interesting that this decrease in uric acid concentrations does not point to the same time frame for transitioning into chronic stress as the immune data. While the immune data do not show significant changes until after eight days of the chronic stress protocol, uric acid significantly decreases within the first four days. This finding emphasizes that different physiological metrics can suggest alternate time frames for when the shift into chronic stress occurs.

### Behavioral effects of chronic stress

Finally, behavioral tests show that birds approached novel objects faster and reduced their activity as they progressed through the stress protocol. Note that a faster approach is unlikely to reflect habituation to the novel object because each bird was exposed to an unexperienced object for each trial, which in an earlier study elicited equivalent neophobic responses for each trial (Fischer & Romero, 2016). Interestingly in this study, object type did have a significant main effect on feed latency. Feed latency when the red dish and ribbon were present elicited similar declines over the course of the protocol whereas responses to the plastic egg did not vary. Importantly, object type did not have a main effect in the perch hopping data. Therefore, we feel that focusing on the overall pattern across all object types reflects the accurate biological response.

Faster approaches and lower activity were contrary to our original prediction, which was that neophobic and anxious behaviors would increase with chronic stress. This prediction was again, based on studies that suggested associations between CORT titers and fear/avoidance behavior (e.g., Korte, 2001; Skórzewska et al., 2006). The reduced neophobic behaviors may be a result of this experiment not inducing increased CORT concentrations, as in these other studies. Similarly, perch hopping frequency decreased through the chronic stress protocol, suggesting that overall activity decreased. Therefore, the faster feed times were not a result of random, flighty activity within the cage, but rather deliberate movements towards the novel object and food dish.

Interestingly, feed latencies during the no-object control trials significantly decreased after the initial trial (Fig. 5, white bars). This suggests that the chronic stress protocol caused the birds to approach their food dish faster in general. This does not lessen the significance of the pattern we see in the neophobia trials during which birds also approach the novel objects faster. Birds still took about seven times longer to feed during the neophobia times relative to the no-object controls. Therefore, the neophobic response did not disappear; birds were simply likely to more quickly approach the food dish regardless of there a novel object was present.

We found a significant interaction between group and perch hopping in the control samples (when no novel object was present) (Fig. 6B). Birds that experienced cage rolling first decreased their activity while those experiencing cage tapping first increased their activity by the end of the chronic stress protocol. It is not clear why order was related to perch hopping, but this kind of interaction effect was not apparent in any of the other physiological or behavioral data.

Although our original hypotheses for the behavioral measurements were based on prior studies connecting CORT and behavior, there is an alternative interpretation arising from the reactive scope model that could explain the decreases. The data suggest that chronic stress induced house sparrows to take additional risk when foraging. Rather than delaying their approach towards a novel, potentially dangerous, object, these birds more readily risked potential harm to forage. In certain circumstances this could be quite dangerous, particularly in the wild where novel objects might not be so innocuous as a colored food dish, ribbon, or plastic egg.

Many behavioral ecology studies have attempted to link CORT to broader indices of behavior, including explorative behavior and foraging activity (e.g., Atwell et al., 2012; Crino et al., 2017). The results of this study indicate that at least in some instances, chronic stress-induced changes to CORT and behavior happen at different time scales. This could be particularly important to consider in field-based studies when the stimuli might be on a much larger context, including urbanization and predator presence. Finally, we found it interesting that during the no-object control trials, females tended to be much more active than males. It is particularly intriguing that this sex difference is absent in the neophobia trials when novel objects are present. While the response to novel objects is not affected by sex, underlying sex differences appear to change baseline activity.

## Conclusion

We have shown that both physiology and behavior can significantly change over the course of a short chronic stress protocol. Most importantly, there are different timescales at which the effects of chronic stress are being reflected in the different indices measured here. Additionally, birds became less neophobic and more active. These results suggest that the overall physiology of the birds is entering a new state, perhaps indicative of “chronic stress.” It is possible that these metrics indicate that these birds are closer to the overload threshold of the reactive scope framework. Interestingly, eight days of the chronic stress protocol did not appear sufficient to significantly alter HPA function, although a longer period of stress may have elicited an effect. Furthermore, these results provide important support for the idea that CORT is not the best indicator of chronic stress, particularly considering its complicated relationship with so many other physiological and behavioral systems. Despite decades of research on stress, we still lack the ability to predict under what circumstances pathological symptoms may develop. We hope that these data provide initial steps towards being able to better interpret results pertaining to the vertebrate stress response and their relation to overall individual animal health and fitness.

##  Supplemental Information

10.7717/peerj.4961/supp-1 Data S1 All combined raw data

## Additional Information and Declarations

### Field Study Permissions

The following information was supplied relating to field study approvals (i.e., approving body and any reference numbers): Massachusetts Division of Fisheries and Wildlife provided approval for this study.

### Data Availability

The following information was supplied regarding data availability: The raw data are provided in the Data S1.
